# Phytochemical analysis, GC–MS profile and determination of antibacterial, antifungal, anti-inflammatory, antioxidant activities of peel and seeds extracts (chloroform and ethyl acetate) of *Tamarindus indica* L

**DOI:** 10.1016/j.sjbs.2023.103878

**Published:** 2023-11-25

**Authors:** Adinath N. Tavanappanavar, Sikandar I. Mulla, Chandra Shekhar Seth, Zabin K. Bagewadi, Mohamed Rahamathulla, Mohammed Muqtader Ahmed, Syeda Ayesha Farhana

**Affiliations:** a Department of Biochemistry, School of Allied Health Sciences, REVA University, Bangalore 560064, India; b Department of Botany, University of Delhi, New Delhi 110007, India; c Department of Biotechnology, KLE Technological University, Hubballi, Karnataka 580031, India; d Department of Pharmaceutics, College of Pharmacy, King Khalid University, P. O. Box 62223, Al Faraa, Abha, Saudi Arabia; e Department of Pharmaceutics, College of Pharmacy, Prince Sattam Bin Abdulaziz University, P.O. Box 173, Al-Kharj 11942, Saudi Arabia; f Department of Pharmaceutics, Unaizah College of Pharmacy, Qassim University, Unaizah 51911, Saudi Arabia

**Keywords:** *Tamarindus indica* L., Seeds, Peel, Antimicrobial activity, Anti-inflammatory activity

## Abstract

*Tamarindus indica* L., is widely used tree in ayurvedic medicine. Here, we aimed to understand the presence of important constituents in seeds and peel of Tamarind fruits and their biological activities. Hence, seeds and peel of Tamarind fruits are used for further extraction process by soxhlet method (chloroform and ethyl acetate solvents). Results suggest that the ethyl acetate extract (seeds) consists of terpenoids (72.29 ± 0.513 mg/g), phenolic content (68.67 ± 2.11 mg/g) and flavonoids (26.36 ± 2.03 mg/g) whereas chloroform extract (seeds) has terpenoids (42.29 ± 0.98 mg/g). Similarly, chloroform extract (peel) has terpenoids (25.96 ± 3.20 mg/g) and flavonoids (46.36 ± 2.03 mg/g) whereas ethyl acetate extract (peel) has terpenoids (62.93 ± 0.987 mg/g). Furthermore, anti-inflammation activity results revealed that the chloroform extract of peel was found to be more effective with IC_50_ of 226.14 µg/ml by protein denaturation analysis and with IC_50_ of 245.5 µg/ml on lipoxygenase inhibition activity. Chloroform extract (peel and seeds) shown better antioxidant activity using DPPH than ethyl acetate extract (peel and seeds). Ethyl acetate extract of seeds showed impressive potency by inhibiting the growth of fungus, *Candida albicans.* Additionally, ethyl acetate extract of seeds showed impressive potency inhibiting the growth of *Escherichia coli* than *Bacillus cereus*. GC–MS analysis shown the existence of diverse set of phytochemicals in each extract. Overall, comparative studies highlight the effectiveness of seeds extracts than peel extracts. Moreover, GC–MS results suggest that the seeds and peel extracts (chloroform and ethyl acetate) contains a wide range of compounds (including flavonoids, isovanillic acid, fatty acids and phenolic compounds) which can be utilized for therapeutic purpose.

## 1 Introduction

In recent times, antimicrobial, anti-inflammatory and anti-oxidative properties having biological molecules extracted from plant sources are gaining much attention (Ain et al., 2023, Bagewadi et al., 2019, Brindhadevi et al., 2023, Kumar et al., 2023, Mitropoulou et al., 2023, Shaukat et al., 2023). For example, anti-inflammatory agents are used to treat COVID-19 as well as neuroinflammation and also have anti-carcinogenic as well as diabetes-control properties (Kumar et al., 2023, Merecz-Sadowska et al., 2023; Perico et al., 2022). Hence, various kinds of plant sources such as *Aerva lanata* flower, *Ficus religiosa*, *Kaempferia rotunda* Linn., *Lavandula dentata* L., *Rosmarinus officinalis* L., *Rumex dentatus* L., *Teucrium multicaule*, *Tripleurospermum limosum* etc., are used for different (medicinal/pharmaceutical) purposes like anti-inflammatory, antioxidant activity, antifertility, antifungal, *in vitro* biofilm inhibition of bacteria, antifungal, antidiarrheal, and so on (Baliyan et al., 2022, Brindhadevi et al., 2023, Chen et al., 2022a, El Abdali et al., 2022, Ersoy et al., 2023, Ibrahim et al., 2022, Khaliq et al., 2023, Sahoo et al., 2023).

Similarly, *Tamarindus indica* tree source is also utilized for medicinal purpose which is a long lived evergreen hardwood tree found in different countries/region, for example, Egypt, Africa (tropical region) and Asia (including India) (Aly et al., 2023, Bhadoriya et al., 2011, Ghaly et al., 2023). *Tamarindus indica* tree yields high amount of green fruits. Once green fruit completely ripens in the tree, the fruit becomes a paste like texture (brown/reddish brown color). And the taste of the fruit will get sweet and sour (Yahia, 2011). Over the years, every part of the tree is traditionally used in ayurvedic medicinal system with nutritional value (Morton, 1958). For example, pulp of tree was used as ayurvedic ingredient for detoxifying skin as well as the body polishing purpose (Morton, 1987). Additionally, paste of Tamarind has anti-microbial properties, because of this reason, the Tamarind paste was frequently used to treat wounds (Gupta et al., 2014). The fruits of Tamarind have most of the essential amino acids as per the report of World Health Organization (Glew et al., 2005, Kuru, 2014).

There are reports on different parts of the Tamarind tree which have shown anti-microbial, anti-viral, anti-venom, anti-diabetic, anti-asthmatic activity, anti-oxidant, anti-venomic, antimalarial, anti-asthmatic, and anti-inflammatory activity (Bhadoriya et al., 2011, Ghaly et al., 2023, Kuru, 2014, Usman et al., 2023). Additionally, the extract of tree parts was also utilized traditionally for treating, diarrhoea, dysentery, helminths infections, abdominal pain, wound healing and so on (Joshi et al., 2023, Kuru, 2014).

From the Tamarind tree and its parts, many active phytochemicals have been isolated (Ghaly et al., 2023, Usman et al., 2023) namely some phenolic compounds like tartaric acid, l-(-)mallic acid; triterpenes such as lupanone and lupeol; oils components such as n-hexacosane, eicosanoic acid, b-sitosterol, octacosanyl ferulate and so on including fatty acids and proteins (Rana et al., 2018). However, not much information available on seeds and peel extracts of Tamarind fruits.

Hence, in the current study, we collected Tamarind fruits and separated seeds and peel. Further, these seeds and peel are used for extraction by soxhlet method using ethyl acetate and chloroform solvents, respectively. The extracts of seeds and peel of Tamarind fruits are used for the determination of qualitative and quantitative analysis of phytochemicals. Gas chromatography-mass spectrometry (GC–MS) study was conducted to detect the bioactive molecules presence in both seeds and peel extracts. Furthermore, we studied antibacterial, antifungal, anti-inflammatory and antioxidant activities of peel and seeds extracts (ethyl acetate and chloroform). Here, our research study will further insight in seeds and peel extracts (ethyl acetate and chloroform) components which will eventually help in additional information to current knowledge of Tamarind fruits. That can be further utilized for treatment purpose.

## 2 Materials and methods

### 2.1 *Tamarindus indica* seeds collection and extraction

*Tamarindus indica* fruits collected from agriculture field in Yatanhalli village (Lat: 15.0066082 and Lon: 75.1448748), Shiggaon taluk, Karnataka, India. The collected fruits are further used to separate seeds. The separated seeds are further washed. Seeds and peel are separated and shade dried. Dried seeds and peels are powdered thoroughly using stone mortal and pestle and were used for further extraction.

Approximately 10 g of fine powdered samples of each seeds and peel are used for extraction by soxhlet method using 200 ml of chloroform and ethyl acetate solvents, respectively (Bagewadi et al., 2019). Rotary evaporator was used to dry the supernatants (containing bioactive molecules) collected from seeds and peel samples and dried samples were stored at 4 °C, till further use.

### 2.2 Biochemical methods

Seeds and peel extracts (chloroform and ethyl acetate) of *Tamarindus indica* fruits were evaluated by standard protocols to detect existence of various phyto-constituents like flavonoidss, alkaloids, saponins, phenols, and so on (Bagewadi et al., 2019, Harborne, 1984).

### 2.3 Quantitative determination of phytochemicals

#### 2.3.1 Total phenolic content

Folin-Ciocalteu reagent was used to quantify the total phenolics content in seeds and peel extracts (chloroform and ethyl acetate) with little modification (Bagewadi et al., 2019, Blainski et al., 2013). Here, we have taken 0.5 ml of each extracts (seeds and peel) in clean and dried test tubes, to this 0.5 ml of Folin-Ciocalteu reagent was added and the reaction mixture was kept for 5 to 8 min (at 25 °C). After that, 2 ml of 7.5 % sodium carbonate solution was added and the solution was makeup to 8 ml with double distilled water and kept for further 2 h. The absorbance was noted at 750 nm. Here, to get a calibration curve, standard compound, gallic acid was used and the total phenolic content was determined as described previously as per following equation, Gallic acid (mg) equivalents/gram of sample (mg GAE /g E) (Bagewadi et al., 2019).

#### 2.3.2 Total flavonoids content

The total flavonoids content in each seeds and peel extracts (chloroform and ethyl acetate) were measured by colorimetric assay with little modification (Bagewadi et al., 2019, Fattahi et al., 2014). In a clean and dried test tubes, 0.5 ml of each extracts (seeds and peel) were added. To this solution, 4 ml of double distilled water was added. The mixed solution was further mixed with 0.4 ml 5 % of sodium nitrite and incubated up to 5 min. After specified time, the solution was mixed with 0.3 ml of 10 % aluminium chloride. At 6 min, the solution was mixed with 2 ml of sodium hydroxide (1 M) solution and the final volume was further diluted with 3.3 ml double distilled water and mixed meticulously without delaying the reaction. At 510 nm, the absorbance was noted. Here, to get calibration curve, standard compound quercetin was used. Total flavonoids content of the extract was determined using an equation, Quercetin (mg) equivalents/gram of sample (mg QuE /g E).

#### 2.3.3 Total terpenoids content

100 mg of each seeds and peel extracts (wi) (chloroform and ethyl acetate) were taken separately and soaked in approximately 9 ml of ethanol for a day and filtered with whatman filter paper. The filtrate was further fractionated with 10 ml of petroleum ether. The separated ether extract sample (s) was pre-weighed glass vials and dried completely (wf). The yield (%) of total terpenoids contents was determined as per the procedure described in Malik (2017).

### 2.4 Anti-inflammatory study

#### 2.4.1 Protein denaturation assay

In brief, in a clean 1.5 ml of centrifuge tubes, the reaction mixture consisted of 1 ml of PBS as well as 50 µl of BSA were added, to this, different concentration of each seeds and peel extracts (chloroform and ethyl acetate) were added individually and also performed similar way with standard solution. All the tubes are incubated for 15 min (at 35 °C). Later, denaturation was induced by keeping at 60 °C in hot water bath for 15 min. At 660 nm, the absorbance was noted in UV–Vis spectrophotometer (Company, Labman UV–Visible Spectrophotometer). Aspirin was used as standard (Leelaprakash and Dass, 2011). The percent of inhibition of protein denaturation was evaluated as described in Revankar et al., (2023).

#### 2.4.2 Lipoxygenase assay

The lipoxygenase activities of seeds and peel extracts (chloroform and ethyl acetate) are determined as described previously (Chen et al., 2009) with little modification. 134 μM of linoleic acid as a substrate and 165 U/ml of enzyme was used final concentration. Test extracts of seeds and peel were dissolved in DMSO (1.6 %). At 234 nm, the absorbance was noted in UV–Vis spectrophotometer (Company, Labman UV–Visible Spectrophotometer). Here, inhibition of enzyme was determined in percentage (%) as per the following equation.

(%)=(X-Y)/X×100

Where X = absorbance measured at 234 nm (without a test sample), and Y = absorbance measured at 234 nm with a test sample.

### 2.5 Antioxidant assay

Seeds and peel extracts (chloroform and ethyl acetate) are used to determine antioxidant activity as described previously with minor modification (Shettar et al., 2023). In a test tube, 1.0 ml of α, α-diphenyl-β-picrylhydrazyl (DPPH) (0.2 mM) was taken. To this, 0.5 ml of different concentrations test samples (Seeds and peel extracts) were added and the also standard solutions having concentrations between 100 and 500 µg/ml were also added. The reaction mixture of each test samples and standards are kept individually at room temperature under dark condition for half an hour. At 517 nm, the absorbance was determined in UV–Vis spectrophotometer (Company, Labman UV–Visible Spectrophotometer). Here, standard compound ascorbic acid was used as specified previously (Shettar et al., 2023, Baliyan et al., 2022). The antioxidant or radical scavenging activity was determined in %. The following equation was used to calculation.

Antioxidantactivity(%)=[(X-Y)/X]×100

Where, X and Y are the absorbance of control and test samples, respectively.

### 2.6 Antimicrobial activity

#### 2.6.1 Anti-fungal assay

Potato dextrose agar (PDA) containing Petri plates were used to grow a fungal pathogen, *Candida albicans*. Freshly grown fungal strain (20 h) was inoculated on PDA. Wells were made using sterile well-borer. Here, for positive control, Itracanozole (10 mg/ml) was used as standard drug and for negative control, DMSO was used. Individual extract of different concentrations like 30 µg, 60 µg and 90 µg per ml are added to the well. All plates were kept at 37 °C for incubation (24 h). The antifungal activity was confirmed by determining the diameter of the inhibition zone formed around the well (Biemer, 1973).

#### 2.6.2 Antibacterial assay

The antibacterial effect was evaluated on gram positive [*Bacillus cereus* (MTCC-1369)] and gram negative [*Escherichia coli* (MTCC-739)] pathogens as described previously (Rajagopalachar et al., 2022) with little modifications. Nutrient agar was sterilized and poured into sterile petri plates. After solidification, the agar plate surface is inoculated by spreading 0.1 ml of overnight grown each pure microbial inoculum. Then well made (5 mm diameter) aseptically at specific distance in the petri plates with a sterile cork-borer. Here, for positive control Ampicillin (100 μg/ml) was used standard drug and for negative control DMSO was used to determine the sensitivity of bacterial culture. Various concentrations of extracts (chloroform and ethyl acetate) of seeds and peel samples are used to test antibacterial activity.

### 2.7 Molecular characterization of seeds and peel extracts (chloroform and ethyl acetate) are carried out using GC–MS method

For the determinations of seeds and peel extract samples, GC–MS analysis was performed with little modifications (Massada, 1976, Mulla et al., 2016b). Here, fused silica used as a closed column and Helium gas was used as mobile phase for separation of active components present in the test samples. An aliquot of each extracts (1 µl) were injected into the GC–MS apparatus. The initial temperature of column programmed from 60 °C. The injector temperature was set at 250 °C and during the process temperature flow was set at the speed of rising 10 °C/min with standard specifications. Final temperature was set to 300 °C. Once it reaches the final temperature, it was holding for 6 min. The identification of components was based on the comparison of their mass spectra with those of NIST mass spectral library (Massada, 1976, Mulla et al., 2016b).

### 2.8 Statistical analysis

All the experimental studies are carried out in triplicates and the results were determined as mean ± SEM. Statistics were performed using GraphPad Prism 8.0 and Microsoft excel 2013.

## 3 Results

### 3.1 Phytochemical analysis

The phytocompound analysis of extracts (chloroform and ethyl acetate solvents) of Tamarind fruit seeds showed the presence of different groups of phyto-components. Ethyl acetate extract (seeds) tested positive for terpenoids, saponins, phenolic compounds and flavonoids. Chloroform extract (seeds) tested positive only for terpenoids (Table 1). Additionally, phytochemical analysis of solvent extract of peel indicated the existence of various types of phytocomponents. Chloroform extract of peel sample tested positive for terpenoids and flavonoids. Ethyl acetate extract of peel sample tested positive only for terpenoids (Table 1).

**Table 1:** Phytochemical analysis of extracts (chloroform and ethyl acetate) of seed and peel samples.

**Extract sample**	**Phytochemical tests**	**Organic solvents**	
		**Chloroform**	**Ethyl acetate**
Seed	Alkaloids	-ve	-ve
	Flavonoids	-ve	+ve
	Glycosides	-ve	-ve
	Phenols	-ve	+ve
	Saponins	-ve	+ve
	Tannins	-ve	-ve
	Terpenoids	+ve	+ve
	Steroids	-ve	-ve
Peel	Alkaloids	-ve	-ve
	Flavonoids	+ve	-ve
	Glycosides	-ve	-ve
	Phenols	-ve	-ve
	Saponins	-ve	-ve
	Tannins	-ve	-ve
	Terpenoids	+ve	+ve
	Steroids	-ve	-ve

(+ve = Present and –ve = Absent).

### 3.2 Estimation of total phytochemical content

**Fig. 1:**
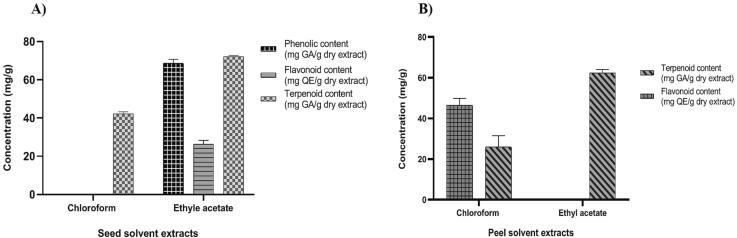
Terpenoid, flavonoids and total phenolic contents of the seeds extracts (A) and peel extracts (B) with respect to the standards. Results mentioned are triplicate study and data expressed in mean ± SEM.

Based on the presence of phytochemicals, their contents were estimated according to their respective standard linear curve. The results of ethyl acetate extract of seeds in accordance with the respective standards shown the presence of high content of terpenoid (72.29 ± 0.513 mg/g), phenolic content (68.67 ± 2.11 mg/g) and flavonoids (26.36 ± 2.03 mg/g) whereas 42.29 ± 0.98 mg/g of terpenoid found in chloroform extract of seeds (Fig. 1a).

Similarly, the results of ethyl acetate extract of peel in accordance with the respective standards shown the presence of high content of terpenoid (62.93 ± 0.987 mg/g) whereas 46.36 ± 2.03 mg/g of Flavonoids content and 25.96 ± 3.20 mg/g of total terpenoids content found in chloroform extract of peel (Fig. 1b).

### 3.3 Anti-inflammatory

Anti-inflammatory activity of extracts (chloroform and ethyl acetate) of seeds and peel were analyzed by very effective protein denaturation protective method and enzyme inhibition lipoxygenase assay.

#### 3.3.1 Protein denaturation

Both seeds and peel extracts showed the concentration dependent anti-inflammatory activity. At increasing concentration, it was observed that ≥ 40 % of anti-inflammatory activity in extracts (chloroform and ethyl acetate) of seeds and peel sample. IC_50_ was calculated accordingly and compared with the standard drug Aspirin (IC_50_ = 83.64 µg/ml) (Table 2a).

**Table 2:** Anti-inflammatory activity of extracts (chloroform and ethyl acetate) of seed and peel samples. Protein denaturation results (IC_50_). B) Lipoxygenase activity results (IC_50_).

**Anti-inflammatory activity**	**Solvents and Std**	**Seed (IC_50_)** µg/ml	**Peel (IC_50_)** µg/ml
**A) Protein denaturation**	Chloroform	225.6	223.1
	Ethyl acetate	270.2	256.1
	Aspirin IC_50_	83.64	
**B) Lipoxygenase activity**	Chloroform	318.6	245.5
	Ethyl acetate	297.1	258.2
	Aspirin IC_50_	96.11	

On comparison of IC_50_ concentrations of seeds and peel extracts, all the extracts showed IC_50_ of > 200 µg/ml concentration which was very much distinguishable from the standard drug (Table 2A).

#### 3.3.2 Lipoxygenase assay

Both seeds and peel extracts (chloroform and ethyl acetate) showed anti-inflammatory potency against lipoxygenase, an inflammatory enzyme. Results suggest that the increasing concentrations of extracts were increases the inhibition level. Chloroform and ethyl acetate extracts of both seeds and peel sample showed more than 50 % inhibition of lipoxygenase enzyme. IC_50_ concentrations of chloroform extract of seeds showed 318.6 µg/ml whereas ethyl acetate extract of seeds showed 297.1 µg/ml. Similarly, IC_50_ concentrations of chloroform extract of peel showed 245.5 µg/ml whereas ethyl acetate extract of peel showed 258.2 µg/ml. All the extracts showed IC_50_ > 200 µg/ml concentration which was very much distinguishable from the standard drug of IC_50_ = 96.11 µg/ml (Table 2B).

### 3.4 Anti-oxidant activity

On assessment of the radical scavenging capacity of extracts (chloroform and ethyl acetate) of seeds as well as peel samples by DPPH method. Both the samples showed a significant radical scavenging ability on comparison with the standard Vitamin-C (Table 3). The inhibitory effect was concentration dependent. The results of DPPH activities are follows, IC_50_ concentrations of chloroform extract of seeds showed 277.4 µg/ml whereas ethyl acetate extract of seeds was 290.2 µg/ml. Similarly, IC_50_ concentrations of chloroform extract of peel showed 283.8 µg/ml whereas ethyl acetate extract of peel showed 306.9 µg/ml (Table 3).

**Table 3:** Anti-oxidant activity of extracts (chloroform and ethyl acetate) of seed and peel samples.

**DPPH activity**		
**Solvents and Std**	**Seed (IC_50_)** µg/ml	**Peel (IC_50_)** µg/ml
Chloroform	277.4	283.8
Ethyl acetate	290.2	306.9
Vitamin C- IC_50_	160.7	

### 3.5 Antifungal activity

**Fig. 2:**
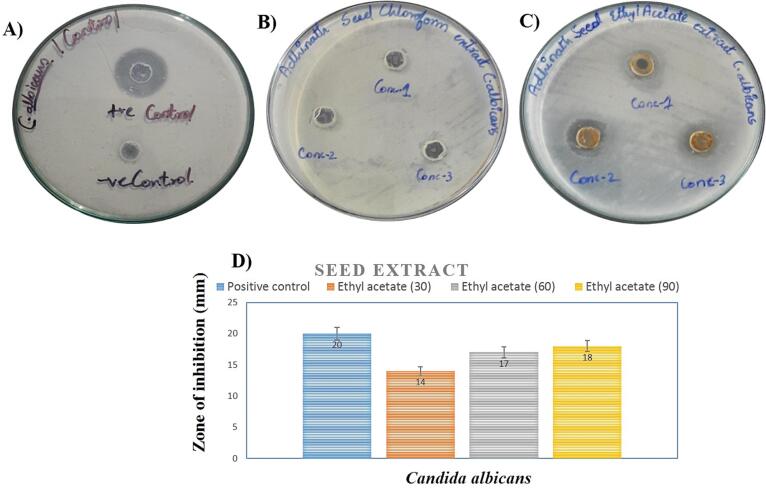
Antifungal activity of seeds extracts (chloroform and ethyl acetate). A) Positive control, B) Seeds chloroform extract, C) Seeds ethyl acetate extract. D) Zone of inhibition of seeds extract and controls. Results mentioned are triplicate study and data expressed in mean ± SEM.

Antifungal activity was studied using 3 different concentrations of each extracts (chloroform and ethyl acetate) of seeds and peel. As compared with the standard drug, only the ethyl acetate solvent extract of seeds showed inhibition against fungus *Candida albicans* with linear increase of concentration (Fig. 2). The zone of inhibition was measured as 14 ± 0.3 mm (30 µg/ml), 17 ± 0.5 mm (60 µg/ml) and 18 ± 0.5 mm (90 µg/mm) with respect to ethyl acetate extract of seeds. However, both solvent extracts of peel are not showed inhibition against *Candida albicans* (data was not shown).

### 3.6 Antibacterial assay

**Fig. 3:**
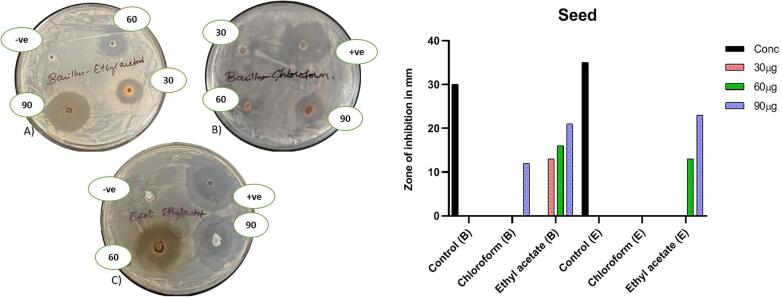
Antibacterial activity of extracts (chloroform and ethyl acetate) of seeds. A) Seeds ethyl acetate extract (Gram + ve), B) seeds chloroform extract (Gram + ve), C) Seeds ethyl acetate extract (Gram -ve). D) Zone of inhibition of seeds extract and controls. In graph* (B) = *Bacillus cereus* and (E) = *E. coli*. Results mentioned are triplicate study and data expressed in mean ± SEM.

Antibacterial potency of three different concentrations of each extracts (chloroform and ethyl acetate) of seeds and peel were assessed against gram positive (*Bacillus cereus*) and gram negative (*E. coli*) pathogens. Positive control, ampicillin showed zone of inhibition measuring about 30 ± 0.7 mm against *Bacillus cereus* and 35 ± 0.2 mm against *E. coli* (Fig. 3) and negative control was not shown inhibition against both pathogens.

The seeds extract was very potent against both the bacteria’s. Against gram positive *Bacillus cereus* bacteria, chloroform extract of seeds showed a zone of inhibition only at 90 µg/ml whereas the ethyl acetate extract of seeds showed a zone of inhibition at all the treated concentrations. Apparently, against the *E. coli,* only the ethyl acetate extract of seeds showed a zone of inhibition parallel to increased concentration (Fig. 3). Extracts (Chloroform and ethyl acetate) of peel has not shown inhibition against *Bacillus cereus* and *E. coli* (data was not shown)*.*

### 3.7 GC–MS profile of extracts (chloroform and ethyl acetate) of seeds and peel samples

GC–MS analysis carried out to determine the presence of important molecules in extracts (chloroform and ethyl acetate) of seeds and peel sample. The extract samples showed a variety of organic compounds belonging to different phytochemical groups. For example, peel chloroform solvent extract consists flavonoids (7,9-Di-Tert-Butyl-1-Oxaspiro(4,5)Deca-6,9-Diene-2,8-Dione, RT-26.401), Saturated fatty acid (Hexadecanoic Acid, RT-27.471), Fatty acids (9,12-Octadecadienoic Acid, RT-32.437; (9z)-Octadecenoic Acid, RT-33.413) etc [Table 4 and Fig. S1, Supplementary information (SI)]. Similarly, seeds chloroform solvent extract consists fatty acid methyl esters (Methyl Palmitate, RT-26.337; Linoleic acid, methyl ester, RT-30.781; Methyl Stearate, RT-31.827), Saturated fatty acids (Lauric Acid, RT-32.361; Eicosanoic acid, methyl ester, RT-37.668), Methyl esters (Methyl Docosanoate, RT- 41.876; Methyl Lignocerate, RT-45.450) etc [Table 5 and Fig. S2 (SI)]. On the other hand, peel ethyl acetate solvent extract consists phenolic compound (Isovanillic acid, RT-21.522), fatty acids (Tetradecanoic acid, RT-25.221; 9-octadecenoic acid (z)-, RT-32.521), Fatty acid Methyl ester (Methyl palmitate, RT-28.358) etc [Table 6 and Fig. S3 (SI)]. Similarly, seeds ethyl acetate solvent extract consists phenol compound (2,4-Ditert-Butylphenol, RT-19.501), fatty acids (1-Tetradecene, RT-16.329; 1-Heneicosanol, RT-31.374), Fatty acid Methyl ester (Linoleic Acid Methyl Ester, RT-31.576) etc [Table 7 and Fig. S4 (SI)].

**Table 4:** GC–MS compounds profile of peel chloroform solvent extract.

**Peak No.**	**Peel chloroform extract compound names**	**Retention time (RT)**	**Base *m*/*z***	**Nature**
1	Neophytadiene	24.549	68.10	Alkenes and Diterpenes
2	Hexa hydrofarnesylacetone	24.705	58.05	Ketones
3	7,9-Di-Tert-Butyl-1-Oxaspiro(4,5)Deca-6,9-Diene-2,8-Dione	26.401	57.10	Flavonoids
4	2-(2-Hydroxyethylmercapto)Benzothiazole	26.874	167.00	Ethanol
5	Hexadecanoic Acid	27.471	73.05	Saturated fatty acid
6	1-Phenyl-2-Pyrazoline	29.417	146.10	Phenyl
7	9,12-Octadecadienoic Acid	32.437	67.05	Fatty acid
8	(9z)-Octadecenoic Acid	33.413	57.10	Fatty acid
9	Hexadecanamide	33.915	59.05	Fatty amide
10	Octadecanamide	39.157	59.05	Fatty amide
11	Eicosane	41.240	57.10	Alkanes
12	1,2-Benzenedicarboxylic Acid	42.267	149.05	aromatic dicarboxylic acid
13	Tetracosane	43.070	57.05	Alkanes
14	1,1-Dichloro-2,2,3,3-Tetramethylcyclopropane	44.037	131.10	Methyl alkanes
15	Ritetracontane	44.798	57.10	Alkanes
16	1,4-Benzenedicarboxylic Acid, Bis(2-Ethylhexyl) Ester	45.828	70.10	Di-ester

**Table 5:** GC–MS compounds profile of seed chloroform solvent extract.

**Peak No**	**Seed chloroform extract compound names**	**Retention time**	**Base *m*/*z***	**Nature**
1	Methyl Palmitate	26.337	74.05	Fatty acid Methyl ester
2	Linoleic Acid, Methyl Ester	30.781	67.10	Fatty acid Methyl ester
3	Elaidic Acid Methyl Ester	30.948	55.05	Unsaturated trans fatty acid
4	Methyl Stearate	31.827	74.05	Fatty acid Methyl ester
5	Lauric Acid	32.361	85.10	Saturated fatty acid
6	1-Hydroxy-2,2,6,6-Tetramethyl-3-Piperidinomethyl-4-Piperidone	37.345	98.10	Hydroxyl amine
7	Eicosanoic Acid, Methyl Ester	37.668	74.10	Saturated fatty acid
8	3-Cyclopentylpropionic Acid, 2-Dimethylaminoethyl Ester	40.426	58.05	Aliphatic carboxylic acid
9	Methyl Docosanoate	41.876	74.05	Methyl ester
10	Linoleic Acid Chloride	44.855	55.05	Fatty acid chloride
11	Methyl Lignocerate	45.450	74.05	Methyl ester
12	Dioctyl Terephthalate	45.819	70.10	Ester

**Table 6:** GC–MS compounds profile of peel ethyl acetate solvent extract.

**Peak No.**	**Peel ethyl acetate extract compound names**	**Retention time**	**Base *m*/*z***	**Nature**
1	Dimethyl dl-malate	9.619	103.10	Dimethyl ester
2	Isovanillic acid	21.522	168.10	methoxybenzoic acids
3	Tetradecanoic acid	25.221	73.10	Fatty acid
4	Hexahydrofarnesyl acetone	26.742	58.05	Ketones
5	Methyl palmitate	28.358	74.10	Fatty acid Methyl ester
6	Hexadecanoic acid	29.291	73.10	Saturated fatty acid
7	Ethyl palmitate	29.674	88.10	Ethyl ester
8	Linoleoyl chloride	31.690	55.05	Fatty acid chloride
9	9-octadecenoic acid (z)-	32.521	55.05	Fatty acid

**Table 7:** GC–MS compounds profile of seed ethyl acetate solvent extract.

**Peak No.**	**Seed ethyl acetate extract compound names**	**Retention time**	**Base *m*/*z***	**Nature**
1	1-Tetradecene	16.329	55.05	Fatty acid
2	2,4-Ditert-Butylphenol	19.501	191.20	Phenol
3	1-Hexadecene	21.201	55.05	Alkenes
4	E-15-Heptadecenal	25.611	55.05	Phenol
5	Ethyl Palmitate	29.665	88.10	Ethyl ester
6	1-Heneicosanol	31.374	55.05	Fatty acid
7	Linoleic Acid Methyl Ester	31.576	67.10	Fatty acid Methyl ester
8	10-Octadecenoic Acid, Methyl Ester	31.675	55.05	Methyl ester
9	Ethyl Linoleate	32.780	67.10	Fatty acid ethyl ester
10	Ethyl Stearate	33.320	88.10	Stearic acid ethyl ester
11	Heptadecyl Acetate	33.590	55.05	ester

## 4 Discussions

Presently, reports suggest that the several groups of microorganisms are shown resistant towards antibiotics and antimicrobial agents and also some of the organisms have capacity to degrade such medicines (Chibwe et al., 2023, Mulla et al., 2016a, Mulla et al., 2016b, Mulla et al., 2018, Wang et al., 2023a, Wang et al., 2023b, Zarzecka et al., 2022). It is because of their presence in the environment either through excretion by animals as well as human beings and/or through incomplete removal from the wastewater treatment systems (Golgeri et al., 2022, Mulla et al., 2016c, Mulla et al., 2020). For example, antimicrobial agents like triclosan and triclocarban are degraded by microorganisms (Mulla et al., 2016a, Mulla et al., 2016b, Mulla et al., 2020). Similarly, sulphonamide drugs like sulfadiazine, sulfamethazine and sulfamethoxazole are also among the antibiotics and were degraded individually by various types of microorganisms (Mulla et al., 2016c, Mulla et al., 2018, Mulla et al., 2023, Wang et al., 2023a). Moreover, there are reports on medicines like ciprofloxacin, tetracycline, levofloxacin (Fluoroquinolone), carbamazepine and diclofenac were degraded/removed by microorganisms (Ben Ayed et al., 2022, Chen et al., 2022b, Shah et al., 2022, Wang et al., 2023b, Wojcieszyńska et al., 2023). Hence, various researchers are looking alternative ways like plant based molecules to control such organisms’ growth. Additionally, it was observed that biomolecules extract from plant sources are useful in industrial purpose.

Tamarind fruits is a household fruit of Asian countries (including India). Maximum south Indian cuisines involve the usage of tamarind fruit and plant young leaves (Rao and Mathew, 2012). Even the Indian Ayurveda uses all the major parts of the plant to cure various human diseases. Based on these data, in the current study we have selected the seeds and peel of Tamarind fruits plant parts to look their biological activities. The selected seeds and its peel are extracted using solvent systems (chloroform and ethyl acetate). The extracts were analyzed for the presence of diverse phytochemicals. In both the samples extracts, presence of phenolic compounds, flavonoids and terpenoids were observed. Further total phenolic, flavonoids and terpenoid contents were determined based on the presence of the phytochemicals according to their solvent extracts. Subsequently, in the seeds ethyl acetate extract, terpenoid content was found highest (72.20 ± 0.51 mg/g) on comparison with the seeds chloroform extract and peel solvent extracts. Whereas total phenolic content was estimated only in seeds ethyl acetate extract based on its presence and was found to be 68.67 ± 2.11. However, total flavonoids content was found to be significantly high (46.36 ± 2.03) in peel chloroform extract than the seeds ethyl acetate extract. Similarly, previous studies on screening of phytochemicals in the different parts of the tamarind tree also have shown specific results (Abdelrahman and Mariod, 2019, Roy et al., 2020, Sookying et al., 2022).

With the hint of vast content of major phytochemicals presence, seeds and peel samples were analyzed for its bioactivities. Primarily they were tested against inflammation using protein denaturation and lipoxygenase assay. Here, protein denaturation was done using heat induction. Heat-denatured proteins exhibit antigens linked to type III hypersensitivity reactions (Johansen et al., 2005). Denatured proteins are one of the main reasons of inflammation (Silvestrini and Silvestrini, 2022). Because of this sever inflammation in the human system, all the major interactions of the protein abruption lead to protein denaturation. Many non-steroidal anti-inflammatory drugs show protection towards protein denaturation (Kpemissi et al., 2023). Hence, this parameter was considered to evaluate the anti-inflammatory effect of the two samples. On analysis, the anti-inflammatory activity was found to be dependent on increasing concentration among all the concentration (Table 2). The data was compared for its effectiveness using the standard anti-inflammatory drug, aspirin. In association with the IC_50_ in protein denaturation assay of the extracts (chloroform) of peel was found to be effective as it showed a prominent inhibition of protein denaturation in its lower concentration. Even though it was distinguishable from the standard drug. Many other plant extracts have also shown the similar kind of anti-inflammatory effect (Anokwah et al., 2022, Dharmadeva et al., 2018).

Lipoxygenase in the human body is mainly involved in stimulation of the inflammation. It’s been linked to many prominent inflammatory disorders (Mashima and Okuyama, 2015). Prostaglandins and leukotrienes are synthesized by lipoxygenase (Cook et al., 1993), and so they are linked to disease development, and their inhibition is regarded as an important step in disease prevention (Hu and Ma, 2018). Hence, inhibition of inflammatory enzyme lipoxygenase is considered for the study. On analysis, extracts (chloroform and ethyl acetate) of both seeds and peel samples showed prominent inhibition of lipoxygenase enzyme in concentration dependent manner. On consideration of IC_50_ values of the solvents of two samples highlight the potency of peel than seeds sample. The results from these samples which was found to be evident with the previous similar work (Chung et al., 2009, Lončarić et al., 2021, Muleya et al., 2017).

As the inflammation in interlinked with oxidative stress and damage, the extracts were explored for its anti-oxidant activity (Biswas, 2016). A stable radical was generated using DPPH, and the radical scavenging ability was accessed. On analysis, extracts (chloroform and ethyl acetate) of seeds and the peel samples depicted very significant anti-oxidant activity. Results suggest that the seeds extracts have better antioxidant activity than peel extracts. There are reports on different parts of the plant source extracts are used to study antioxidant activity and found good results (Natukunda et al., 2015, Sandesh et al., 2014). Hence, plant based extracted molecules have advantage over chemically synthesized molecules due their non-toxicity.

Anti-fungal activity was tested mainly against, *Candida albicans*. It is a most common human fungal pathogen. Hence, in our present study, extracts of both seeds and peel samples were tested against *C. albicans* growth. Among the seeds and peel samples, ethyl acetate extract of seeds showed better zone of inhibition (18 mm ± 0.5) at higher concentration of (90 µg/ml). On the other hand, there is report on silver nanoparticles synthesis carried out using root extract of *Furcraea foetida* showed a zone of inhibition (16 mm ± 2) at 100 µg/100 µl against *C. albicans* (Sitrarasi et al., 2022). Similar kind of results also observed previously (Essghaier et al., 2022; 38 Liu et al., 2017). However, extracts of peel had no effect on inhibition of *Candida albicans*. It’s been evident from the results that only the seeds had anti-fungal effect against *C. albicans.*

Furthermore, on screening for anti-bacterial potency between the seeds and peel extract against gram positive bacteria *Bacillus cereus* and gram negative bacteria *E. coli*. Majority of the pathogenic and drug resistant bacteria are gram negative because of its unique structure (Breijyeh et al., 2020). Hence, structurally and pathologically distinguishable bacterial species were considered for the inhibition study using plant source extracts as well as nanoparticles (Bagewadi et al., 2019, Essghaier et al., 2022, Rajagopalachar et al., 2022) and compared with the standard drugs. Here, both solvent extracts of peel sample had zero effect on both organisms. Contradictorily, seeds chloroform extract at 90 µg/ml concentration and seeds ethyl acetate extract at all three concentration range showed inhibition of *Bacillus cereus* growth. Additionally, against gram negative *E. coli* bacteria only the seeds ethyl acetate extract showed an impressive inhibition whereas the seeds chloroform extract had no effect on the bacteria at all three concentrations. On a note, it was definite that only the seeds ethyl acetate had the significant antibacterial effect.

As the extracts presented impressive bioactivities, the potent extracts from both the samples were explored for their specific phytochemicals using analytical technique like GC–MS. Various researcher studied plant source extraction using different solvents and were further analyzed using chromatographic techniques like GC–MS, UPLC-ESI-Orbitrap-MS, UPLC-ESI-Q-TOF-MS^E^, HPLC etc., to observe the presence of important biomolecules that can be utilized to treat inflammatory, wound healing, antioxidants, antimicrobial activity, hepatoprotective activity and so on (Aly et al., 2023, Chen et al., 2022a, Djebari et al., 2023, Razali et al., 2012, Wang et al., 2022, Youssef et al., 2023). On analysis with GC–MS, it revealed the presence of versatile group of phytocompounds among all the solvent extracts from both the samples. On keen observation of the GC–MS reports, mainly flavonoids, isovanillic acid, fatty acids, fatty acid methyl esters and phenolic compounds are high among all the extracts. The phytochemicals were differed from solvent to solvent showcasing the unique phytoconstituents presence.

## 5 Conclusions

In the current study, the extracts (chloroform and ethyl acetate extract) of seeds and peel samples showed biological activities. Results further revealed that the bioactivities of the samples with different extracts differ based on their phytochemical compositions. Furthermore, peel extracts effectiveness against inflammation was significant. Results are also suggesting that the seeds extracts have significant effectiveness on antioxidant activity. Additionally, on anti-fungal and anti-bacterial potency, seeds extract showed significant inhibition than peel extract. Analytical technique like GC–MS analysis reveals that the presence of various kinds of biomolecules in seed and peel extracts (chloroform and ethyl acetate) of *Tamarindus indica* fruits. Overall, from the study, it can be concluded that, both the samples are potent in specific biological activities which solely depends on their phytochemical compositions that involves in the mechanism of action of that particular activity. Some of the molecules present in both samples extracts are potential drug contenders especially with respect to advancement in medicinal industry.

## Declaration of competing interest

The authors declare that they have no known competing financial interests or personal relationships that could have appeared to influence the work reported in this paper.
