# Supplementary material for: Phytochemical analysis, GC–MS profile and determination of antibacterial, antifungal, anti-inflammatory, antioxidant activities of peel and seeds extracts (chloroform and ethyl acetate) of Tamarindus indica L
Source: Saudi J Biol Sci. 2023 Nov 25;31(1):103878. doi: 10.1016/j.sjbs.2023.103878 (PMC10730893; doi:10.1016/j.sjbs.2023.103878)
Supplement: Supplementary data 1 [file mmc1.docx]

**Supplementary Information**

**Phytochemical analysis, GC-MS profile and determination of antibacterial, antifungal, anti-inflammatory, antioxidant activities of peel and seed extracts (chloroform and ethyl acetate) of *Tamarindus indica* L.**

Total Pages - 04, Number of figures- 04

**Results**


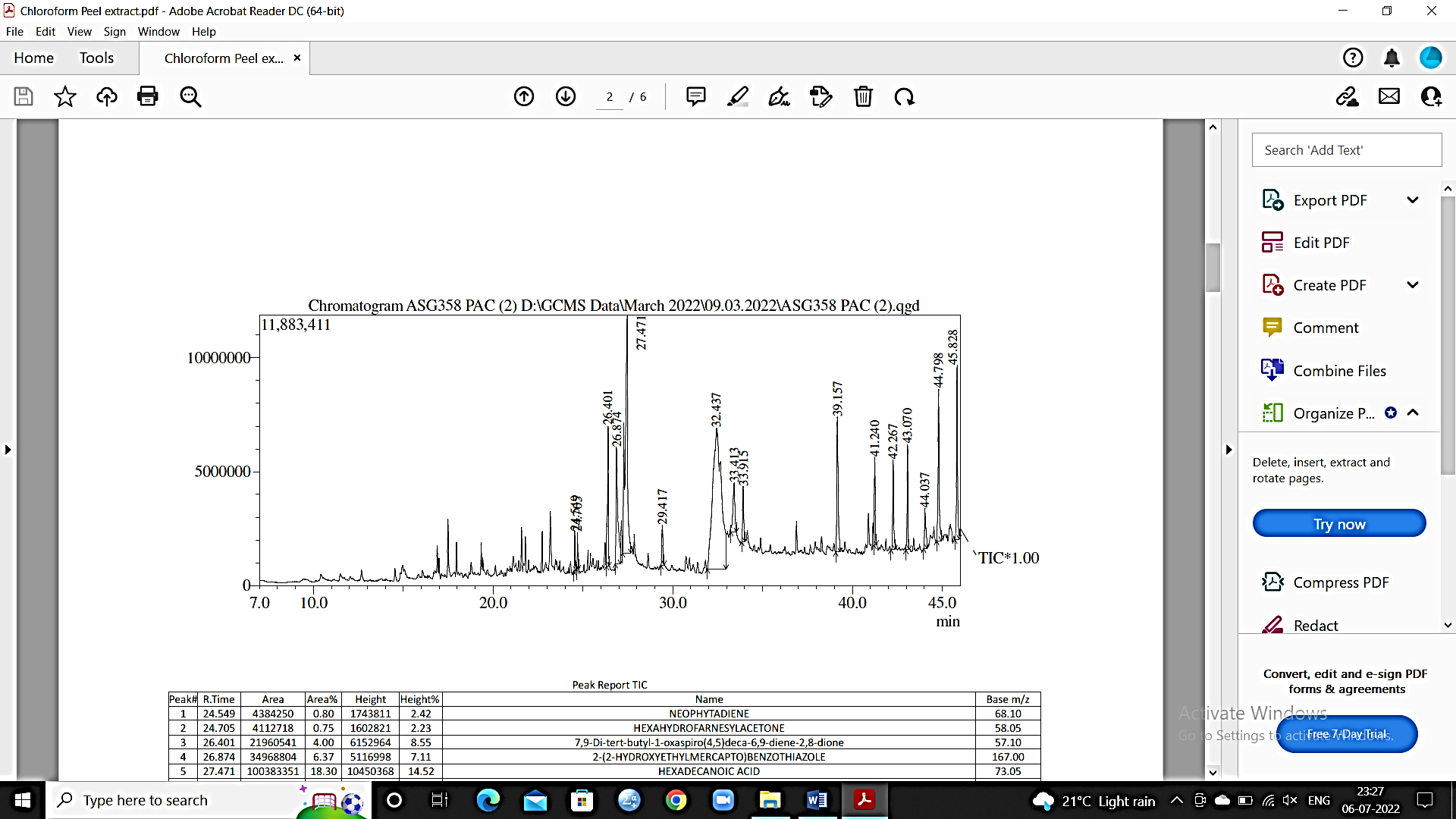


**Fig. S1.** GC-MS chromatogram of peel chloroform solvent extract.


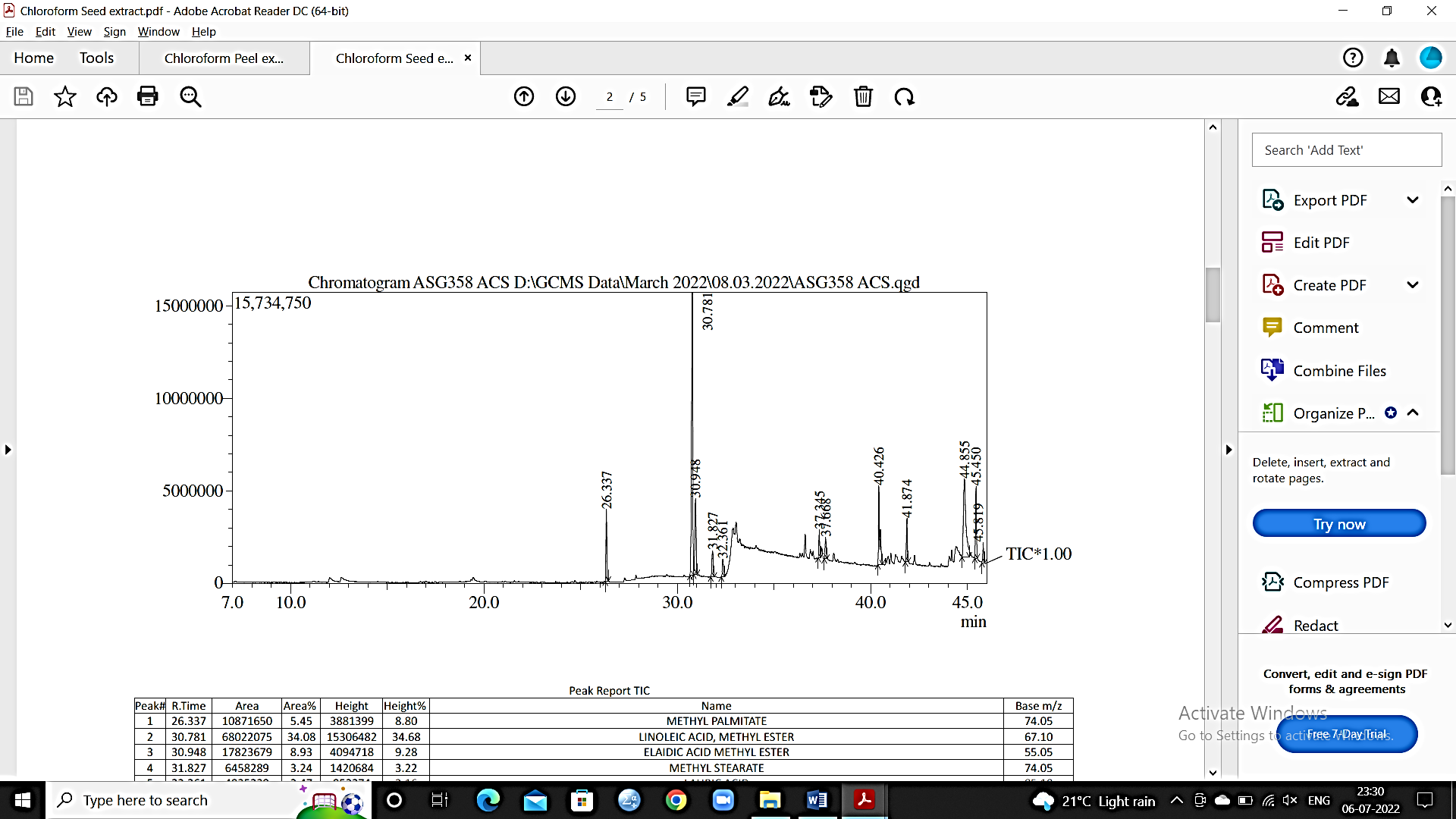


**Fig. S2.** GC-MS chromatogram of seed chloroform solvent extract.


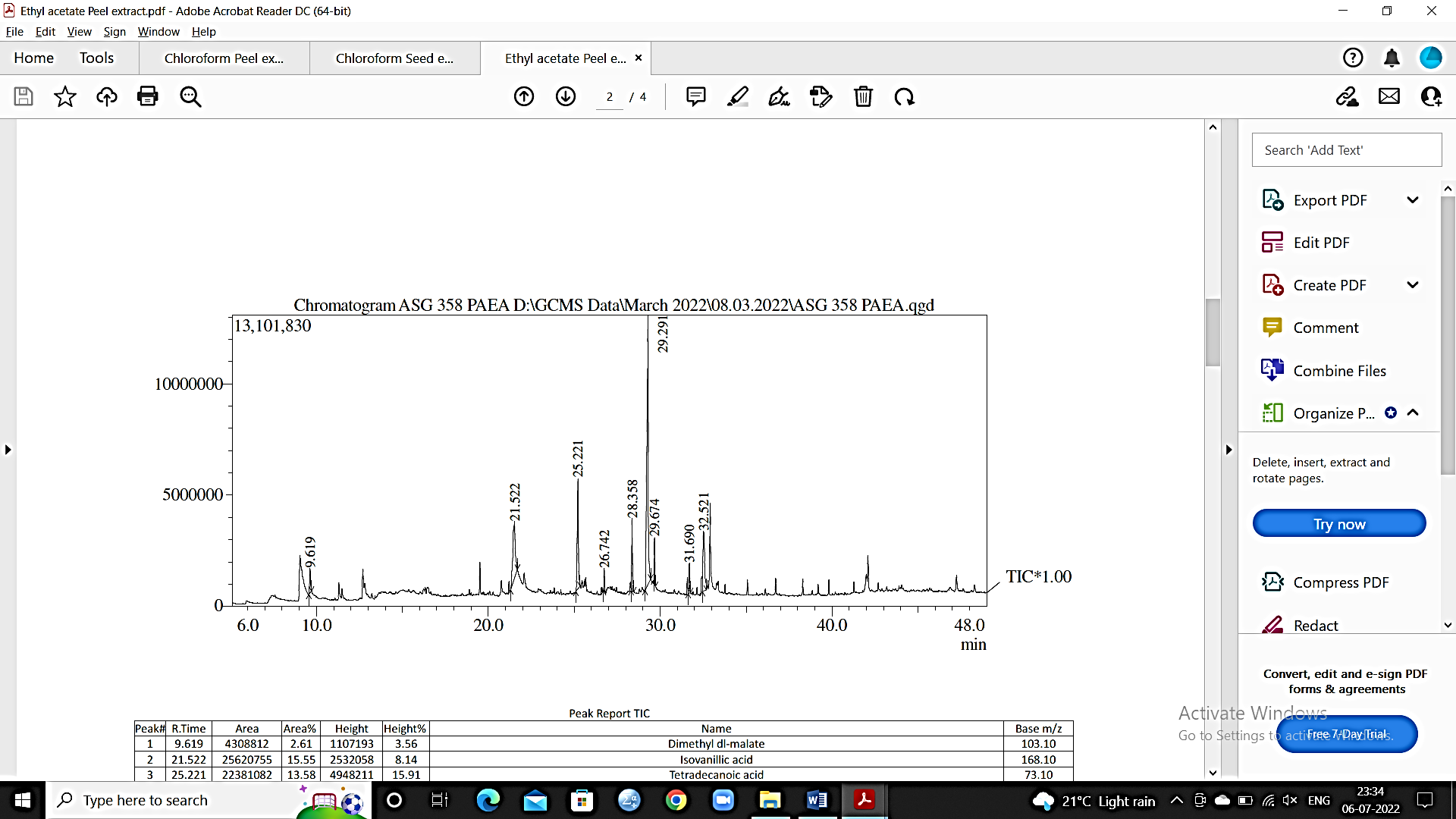


**Fig. S3.** GC-MS chromatogram of peel ethyl acetate solvent extract.


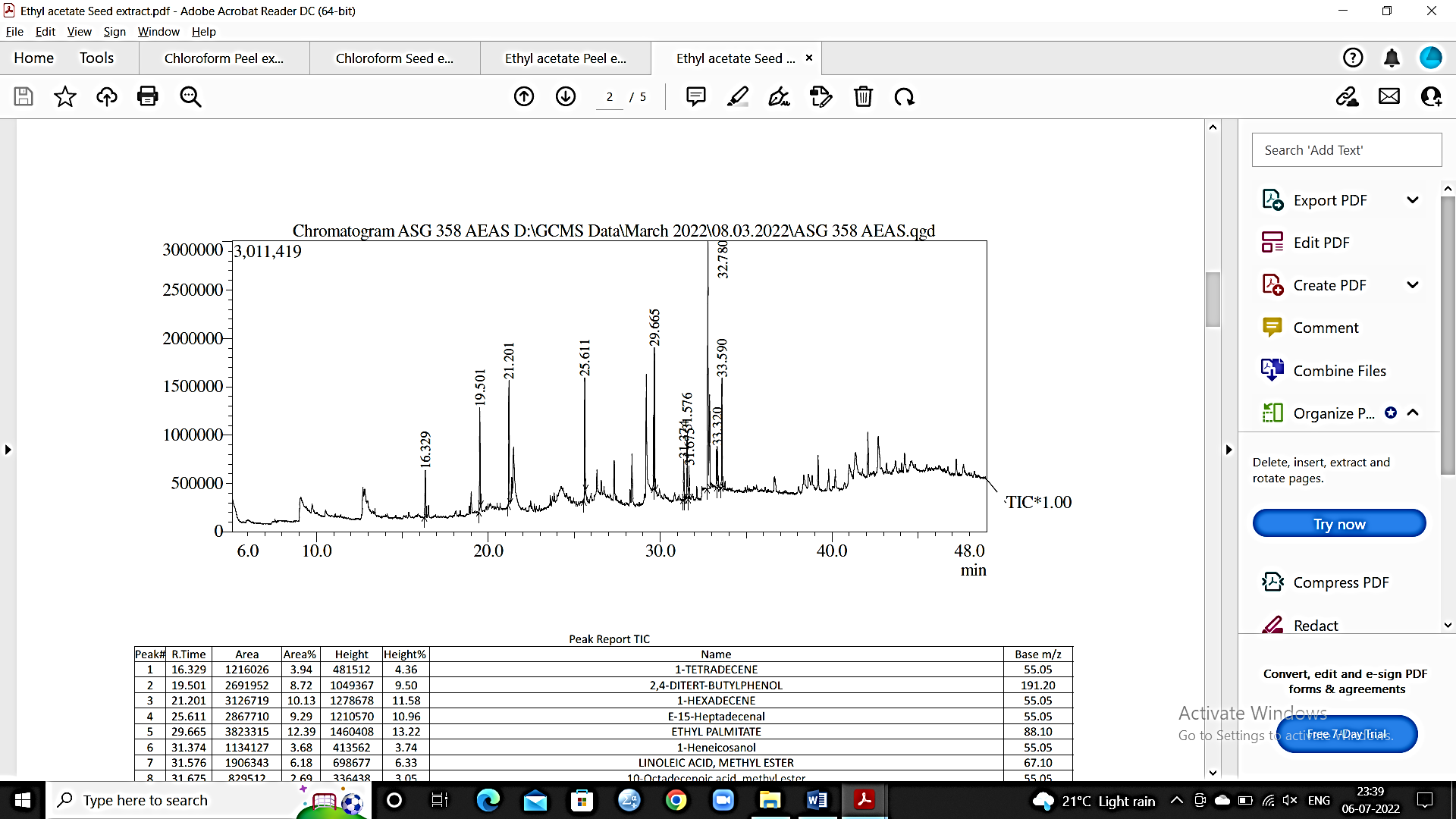


**Fig. S4.** GC-MS chromatogram of seed ethyl acetate solvent extract.
